# Fine Classification of UAV Urban Nighttime Light Images Based on Object-Oriented Approach

**DOI:** 10.3390/s23042180

**Published:** 2023-02-15

**Authors:** Daoquan Zhang, Deping Li, Liang Zhou, Jiejie Wu

**Affiliations:** 1 Hunan Key Laboratory of Geospatial Big Data Mining and Application, Hunan Normal University, Changsha 410081, China; 2 School of Geographic Sciences, Hunan Normal University, Changsha 410081, China

**Keywords:** unmanned aerial vehicle (UAV), object-oriented, machine learning, nighttime urban remote sensing, tilt photogrammetry, urban light classification

## Abstract

Fine classification of urban nighttime lighting is a key prerequisite step for small-scale nighttime urban research. In order to fill the gap of high-resolution urban nighttime light image classification and recognition research, this paper is based on a small rotary-wing UAV platform, taking the nighttime static monocular tilted light images of communities near Meixi Lake in Changsha City as research data. Using an object-oriented classification method to fully extract the spectral, textural and geometric features of urban nighttime lights, we build four types of classification models based on random forest (RF), support vector machine (SVM), K-nearest neighbor (KNN) and decision tree (DT), respectively, to finely extract five types of nighttime lights: window light, neon light, road reflective light, building reflective light and background. The main conclusions are as follows: (i) The equal division of the image into three regions according to the visual direction can alleviate the variable scale problem of monocular tilted images, and the multiresolution segmentation results combined with Canny edge detection are more suitable for urban nighttime lighting images; (ii) RF has the highest classification accuracy among the four classification algorithms, with an overall classification accuracy of 95.36% and a kappa coefficient of 0.9381 in the far view region, followed by SVM, KNN and DT as the worst; (iii) Among the fine classification results of urban light types, window light and background have the highest classification accuracy, with both UA and PA above 93% in the RF classification model, while road reflective light has the lowest accuracy; (iv) Among the selected classification features, the spectral features have the highest contribution rates, which are above 59% in all three regions, followed by the textural features and the geometric features with the smallest contribution rates. This paper demonstrates the feasibility of nighttime UAV static monocular tilt image data for fine classification of urban light types based on an object-oriented classification approach, provides data and technical support for small-scale urban nighttime research such as community building identification and nighttime human activity perception.

## 1. Introduction

Nighttime lighting is a reflection of human activity, economic development and energy use [1,2]. Traditional city night light images are mostly obtained by satellite remote sensing, while small rotary-wing UAVs, as a new remote sensing platform, can provide ultra-high resolution city night light images at the vertical level through tilt photography [3,4,5,6,7]. The distribution of different types of urban lights reflects the internal structure of the city and the intensity of human activities. Therefore, the classification and identification of urban nighttime lights is a key prerequisite step for small-scale nighttime urban research, and the fine classification of various types of urban lights has important research significance and application value for community building identification, urban emergency rescue and nighttime human activity perception.

The study of fine classification of urban night lights needs to consider the extraction of features, the selection of classification methods and the evaluation methods of classification accuracy, among which the most important is the selection of classification methods. The study of fine classification of high-resolution remote sensing images, such as UAVs, mostly adopts an object-oriented classification approach; object-orientation uses the segmented object as the basic unit and can take full advantage of the spectral, geometric and textural features of the object to be classified [8]. In addition, the researcher adds various features according to the characteristics of the object under study, such as adding vegetation indices to identify changes in urban tree cover [9], adding topographic information to classify features [10], etc. Of course, too much feature information can lead to redundancy. Yang K et al. [11] demonstrated that differences in feature dimensionality and importance are the main factors contributing to variation in olive tree extraction accuracy; Guo Q et al. [12] compared different feature combination schemes and showed that the combination by feature elimination had the highest accuracy in urban tree classification. Object-oriented approaches are often combined with machine learning in the selection of classification algorithms. In order to explore the most suitable machine learning algorithm, Cao J et al. [13] compared the accuracies of support vector machine (SVM) and K-nearest neighbor (KNN) algorithms for mangrove species classification, and the result was that SVM was more accurate than KNN; Ye, Z et al. [14] compared the accuracy of random forest (RF), support vector machine (SVM) and K-nearest neighbor (KNN) algorithms in extracting urban impervious surfaces and concluded that RF had the highest extraction accuracy. LIANG, L et al. [15] compared the accuracy of five machine learning algorithms for extracting permafrost hot thaw slip boundaries and concluded that SVM had the highest accuracy; Pádua L et al. [16] compared the fine classification results of support vector machines (SVM), random forests (RF) and artificial neural networks (ANN) for vineyards and concluded that the overall classification performance of ANN was better. It can be seen that the most suitable machine learning algorithms will vary when conducting object-oriented classification studies on different research objects. Additionally, from the viewpoint of the previous research on image time, the research on the combination of object-oriented and UAV images mainly focuses on daytime images, there is less research on the fine classification of city lights at night and there is no research on the fine classification of urban nighttime UAV images using object-oriented approaches.

Therefore, this paper uses a small rotary-wing UAV as a new nighttime urban remote sensing platform, takes the captured static monocular tilted images as the data source, adopts an object-oriented classification method and explores the classification effect of the object-oriented method on urban nighttime lights by comparing the classification accuracy of four machine learning algorithms—random forest (RF), support vector machine (SVM), K-nearest neighbor (KNN) and decision tree (DT)—fills the gap of fine classification research using ultra-high resolution urban nighttime light images captured by UAV, and expects to provide new data and technical references for smaller-scale urban nighttime light research.

## 2. Materials and Methods

### 2.1. Study Site and Data Acquisition

**Figure 1:**
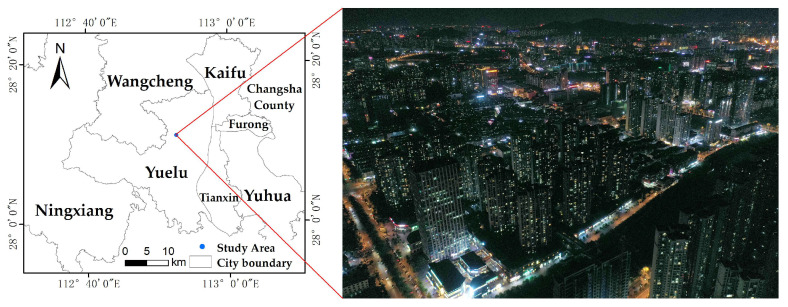
The study area.

The study area is located in Meixihu Street, Yuelu District, Changsha City, Hunan Province, China, mainly including Meixihu Jinmaoyue, Baijiatang, Longqin Bay, Zhenye City and Jiajing, etc. The area has uniform and regular distribution of building height and high community management level, and is a typical urban center community. The images were acquired during the period of 20:00–20:30 at night in May 2022, which is the peak of human nocturnal activity and is representative. Some of the parameters of the UAV were set as shown in Table 1, and the monocular shooting method with fixed vertical takeoff and landing was used to obtain the city night tilt image containing red, green and blue bands (Figure 1). A clear time without wind and clouds was chosen for the shooting to ensure the accuracy.

### 2.2. Research Methods

**Figure 2:**
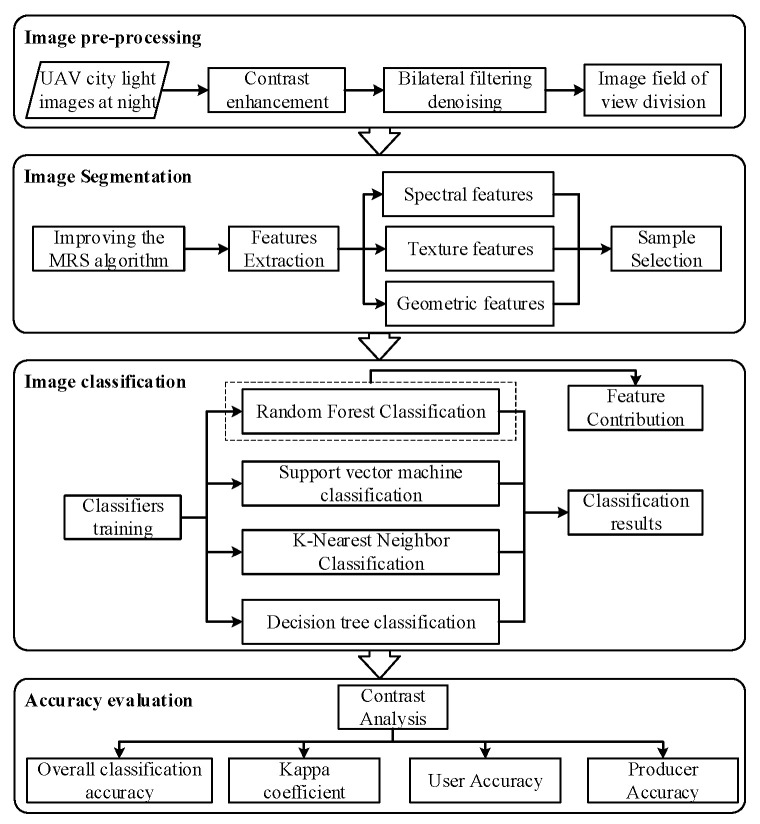
UAV nighttime city light image classification flow chart.

In this study, a classification method of urban night lights based on static monocular tilted UAV visible images is proposed which uses object-oriented approaches and machine learning algorithms. The classification process mainly consists of the following four steps (Figure 2): (1) pre-processing of UAV images; (2) image segmentation and extraction of feature information; (3) image classification, using RF, SVM, KNN and DT classifiers to classify urban nighttime lights; (4) classification accuracy evaluation, selecting the four indexes of OA, kappa coefficient, UA and PA to evaluate the classification accuracy and analyze the accuracy of different machine learning algorithms and light types.

#### 2.2.1. Image Pre-Processing

From Figure 1, it can be seen that the urban nighttime UAV images mainly included five types of lights: window light, neon light, road reflective light, building reflective light and background, and compared with the daytime images, the nighttime UAV images had problems such as low contrast and noise pollution in the dark light areas of the images. In addition, the spatial resolution of the image field of view direction was constantly changing due to the angle of monocular tilt image capture. For the above problems, three pre-processing operations, namely contrast enhancement, bilateral filtering denoising and view field division, were used to solve them.

(1) Contrast enhancement

To solve the problem of low contrast, this paper used the image enhancement module in the PIL library to pre-process the images, such as for contrast enhancement. The process is as follows: first, the original image is converted to grayscale and the arithmetic mean of its grayscale is found; then, a new image is created with the same size and number of channels as the original image, and the value of each channel for each pixel of the new image is the grayscale mean calculated earlier; finally, the new image is created by merging the original image and the new image, and the parameter a determines the weight of the two when they are merged.

(1) $$img_{new}=img1*(1.0-a)+img2*a$$

where img1 is the converted grayscale image, img2 is the original image and $img_{new}$ is the new image after enhancement. a is the ratio that controls the two images when they are combined; when a < 1, the image contrast is reduced; when a = 1, the image remains unchanged; and when a > 1, the image contrast is enhanced.

(2) Bilateral filtering denoising

Bilateral filtering is a nonlinear filter based on the principle of representing the intensity of a pixel by a Gaussian-weighted average of the luminance of the surrounding pixels, making it effective not only in removing image noise but also in better preserving the edge information of the object [17]. Its application in low luminance environments has been demonstrated [18]. In this paper, after several comparison tests, a 50*50 window was finally used for bilateral filtering of the captured urban nighttime UAV images. The processed images effectively removed noise and retained the edge information of various types of lights intact, which was more conducive to the later image segmentation and classification operations.

(3) View field division

**Figure 3:**
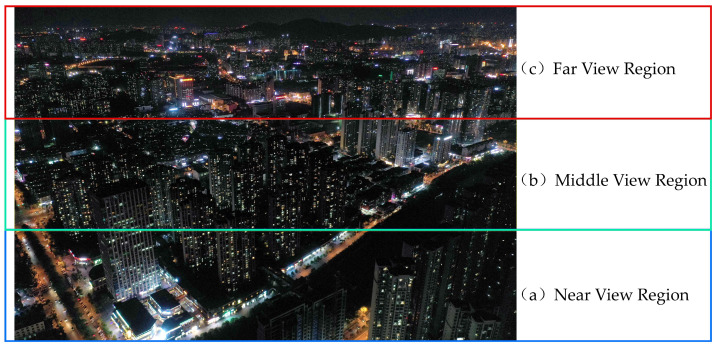
Equally divide the image into near, middle and far images along the direction of the field of view.

Static monocular tilt image is a single-view tilt image taken at a certain height using a single-lens UAV with a fixed vertical takeoff and landing, which has the problem of continuous variable scale, where the spatial resolution of the image becomes gradually smaller along the direction of the field of view and the size of the same type of light in the image varies very much, which has a certain impact on image segmentation and classification. Therefore, in this paper, before image segmentation, the images were equally divided into three images in the near, middle and far view regions according to the view direction (Figure 3), which effectively alleviated the continuous variable scale problem. The subsequent image segmentation and classification operations were based on the near, middle and far view regions.

#### 2.2.2. Multiresolution Segmentation

Image segmentation is a key step in object-oriented classification, and the degree of segmentation directly affects the subsequent classification results. In this paper, the multiresolution segmentation (MRS) algorithm in eCognition 9.0 software was used for image segmentation operation. Multiresolution segmentation is a bottom-up iterative algorithm based on region merging under different scale conditions with a certain image element as the starting point, and the final segmentation objects follow the principle of maximum local heterogeneity among them. The parameters to be set in the multiresolution segmentation process include band weights, scale parameters, shape factors and compactness, among which the scale parameters are mainly determined with the aid of an ESP2 plug-in, which reflects the homogeneity of the segmentation results by introducing the local variance (LV) index and finds the potential optimal segmentation scale by rate of change (ROC) [19].

The light types, such as window lights, in urban nighttime UAV images as small targets are prone to over-segmentation using multiresolution segmentation directly [20,21,22]. In this paper, we finally chose to combine Canny edge detection to alleviate the over-segmentation phenomenon. As a comprehensive algorithm with good performance, Canny edge detection has been tried by many scholars in combination with multiresolution segmentation algorithm [23,24], and the final segmentation results can effectively alleviate the over-segmentation phenomenon and the edge information of the segmented object is more consistent with the real object edge contour.

#### 2.2.3. Features Extraction

Based on the image segmentation results, the spectral, textural and geometric features of the lighting object were extracted. According to the characteristics of different types of night lighting objects, this paper extracted a total of 24 kinds of feature information. Spectral features include mean, standard deviation, maximum brightness difference and brightness in the visible band; textural features include correlation, homogeneity, contrast, standard deviation, angular second moment, dissimilarity, entropy and mean value extracted using gray level co-occurrence matrix (GLCM); geometric features include area, length/width, compactness, density, main direction, roundness, shape index and asymmetry of the lighting object. In order to facilitate subsequent writing, each feature was added with a number for simplified representation, and the specific feature information is shown in Table 2.

#### 2.2.4. Sample Selection

In this paper, based on the three regions divided, different sample sizes were selected according to the actual distribution of each category of light, and a total of 2440 samples were selected. Since there was no road reflective light in the far view region, only the four light types other than road reflective light were available for sample selection. The selected sample data were normalized before model training. To ensure the stability of the classification model and prevent overfitting, a stratified sampling method was used to randomly divide each category of light sample into training and validation sets in the ratio of 7:3 (Table 3).

#### 2.2.5. Classifier

In this paper, based on the synthesis of previous classification algorithms used in object-oriented classification and combined with the lighting objects in this study, we finally determined that four classification algorithms, namely random forest, support vector machine, K-nearest neighbor, and decision tree, were used for comparative analysis to find the most suitable classification algorithm for UAV nighttime urban lighting objects.

Random forest (RF) is a supervised classification algorithm based on integrated learning [25], which is constructed by combining multiple decision trees. The final classification result is obtained by averaging or voting the results of each decision tree. The random forest model has the advantages of high accuracy, robustness and resistance to overfitting, and has better performance in dealing with high-dimensional nonlinear classification problems and is widely used in high-resolution image classification [26,27,28].

Support vector machine (SVM) is a supervised machine learning algorithm based on statistical learning theory with powerful nonlinear and high-dimensional processing capability and high recognition accuracy for small samples [29,30]. The core idea of SVM is to map low-dimensional space to high-dimensional space through kernel function, search for the optimal separation hyperplane in high-dimensional space, maximize the distance between samples and this plane, and thus realize sample classification [31].

The K-nearest neighbor algorithm (KNN) is a nonparametric classification algorithm. The KNN algorithm classifies data based on the nearest or adjacent training examples in a given region, and for a new input, its K-nearest neighbor data are computed, and the majority type of its nearest neighbor data determines the classification of the new input [32]. The K-nearest neighbor algorithm is a simple but highly accurate lazy learning algorithm.

Decision tree (DT) is a supervised learning algorithm that uses a tree structure to construct a classification model, where each internal node of a decision tree is a set of category attributes [33]. DT has the advantages of fast computing speed and high accuracy and can work effectively on relatively small data sets [34], making it widely used in remote sensing image classification.

In this paper, we used Python 3.9 as the runtime environment for machine learning models. When modeling the classifiers, all used the ten-fold cross-validation method to ensure the accuracy of the models when training, and the grid search method was used to select the best parameters for each type of model.

#### 2.2.6. Accuracy Evaluation Methods

In order to accurately analyze the classification accuracy of different machine learning models for UAV nighttime city light images, this paper used the calculation of confusion matrix and employed overall accuracy (OA), kappa coefficient, producer accuracy (PA) and user accuracy (UA) as quantitative metrics to evaluate the classification results.

(2) $$OA=\frac{\sum_{i=1}^{n}T_{ii}}{T_{sum}}\times 100\%$$

(3) $$Kappa=\frac{T_{sum}\sum_{i=1}^{n}T_{ii}-\sum_{i=1}^{n}T_{i+}T_{+i}}{T_{sum}{}^{2}-\sum_{i=1}^{n}T_{i+}T_{+i}}$$

(4) $$PA=\frac{T_{ii}}{T_{+i}}\times 100\%$$

(5) $$UA=\frac{T_{ii}}{T_{i+}}\times 100\%$$

where $T_{sum}$ is the total number of samples, $T_{ii}$ is the number of correctly classified samples, *n* is the total number of categories, $T_{i+}$ is the predicted number of samples in category i, and $T_{+i}$ is the number of true samples in category i.

## 3. Results

### 3.1. Comparison of Segmentation Results

**Figure 4:**
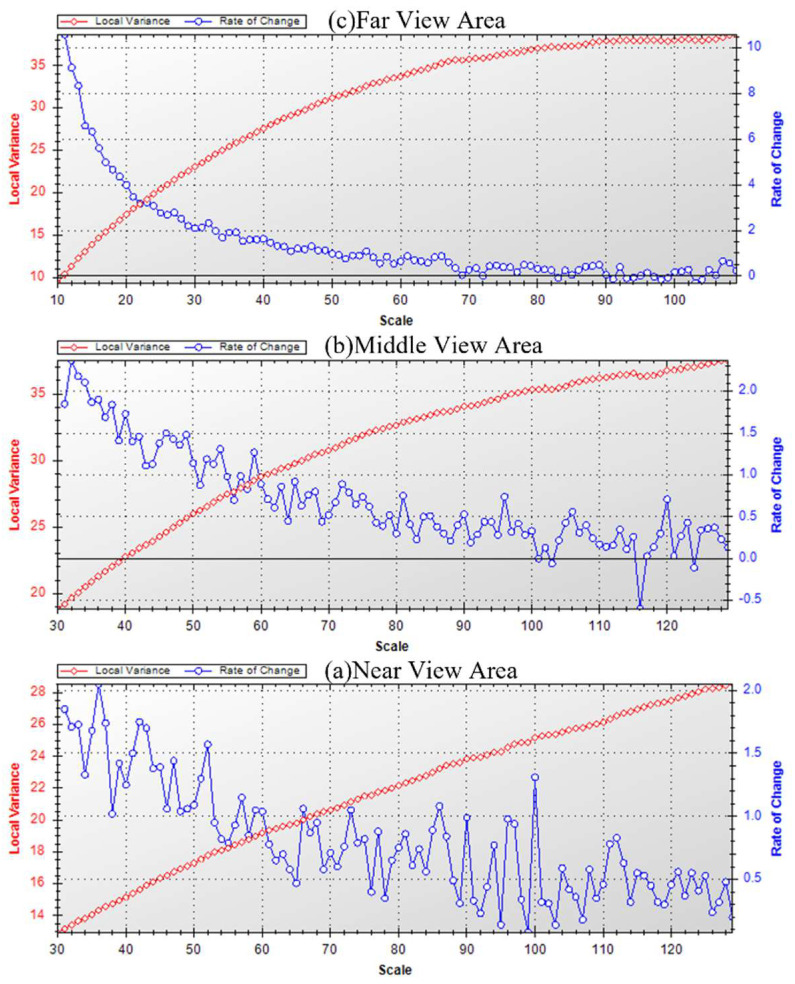
ROC−LV diagrams for near, middle and far view regions.

In this study, the band weights were set to one. The specific segmentation process was as follows: firstly, keeping the scale parameter constant, the shape factor and compactness were segmented in steps of 0.1 and iterated through 0.1–0.9 for multiple segmentation experiments to determine the optimal parameters of both; secondly, the ESP2 plug-in was used to determine the potentially suitable scale parameter (Figure 4), and the final scale parameters were determined by visual interpretation; finally, the optimal segmentation parameter settings for different regions were obtained (Table 4).

**Figure 5:**
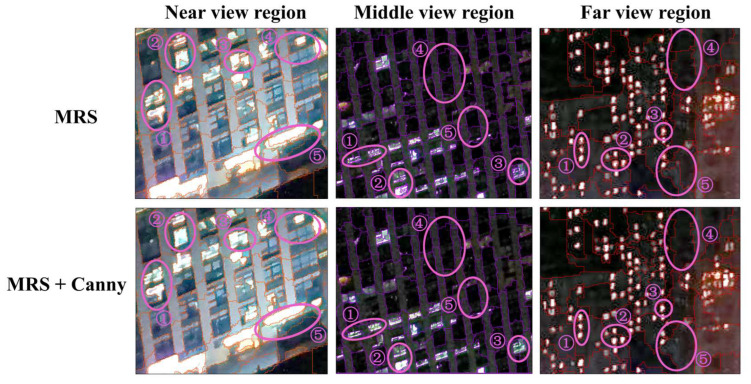
Comparison of segmentation results. (The selected area ① ② ③ indicates the splitting effect of the Window Light, and the selected area ④ ⑤ indicates the split effect between the Building Reflective Light and the Background).

In this paper, we incorporated Canny edge detection results based on multiresolution segmentation to alleviate the over-segmentation phenomenon of some lighting types. From comparing the segmentation results before and after combining Canny edge detection (Figure 5), it was difficult to segment all light types perfectly using only the multiresolution segmentation algorithm, and there was over-segmentation of a window light into multiple window lights (e.g., in the near view region of Figure 5 using only the area marked by circle ①, ② and ③ in MRS), the existence of a halo around the window light (e.g., in the near view region of Figure 5 using only the area marked by circle ④ in MRS) and over-segmentation of the building reflective light and background into multiple objects (e.g., in the middle view region of Figure 5 using only the area marked by circle ④ and ⑤ in MRS). The Canny edge detection can detect the edge contours of all kinds of lights with a low error rate and as much as possible, so the combination of the two makes the extraction results more consistent with the real contours of all kinds of lights. As can be seen from the selected ① ② ③ region in Figure 5, after combining Canny, the over-segmented window light was re-segmented into a complete window light, making the interior of individual window lights more complete and more homogeneous. Additionally, from the ④ ⑤ region selected in Figure 5, we can see that after the combination of the two, the segmentation result of the building reflective light and background was more regular and smoother, which is more in line with the actual situation. The segmentation results in this paper further validate the feasibility of the combination of the two types of algorithms for the application of UAV nighttime urban lighting images.

### 3.2. Classification Results

#### 3.2.1. Results of Fine Classification of Urban Nighttime Lighting

**Figure 6:**
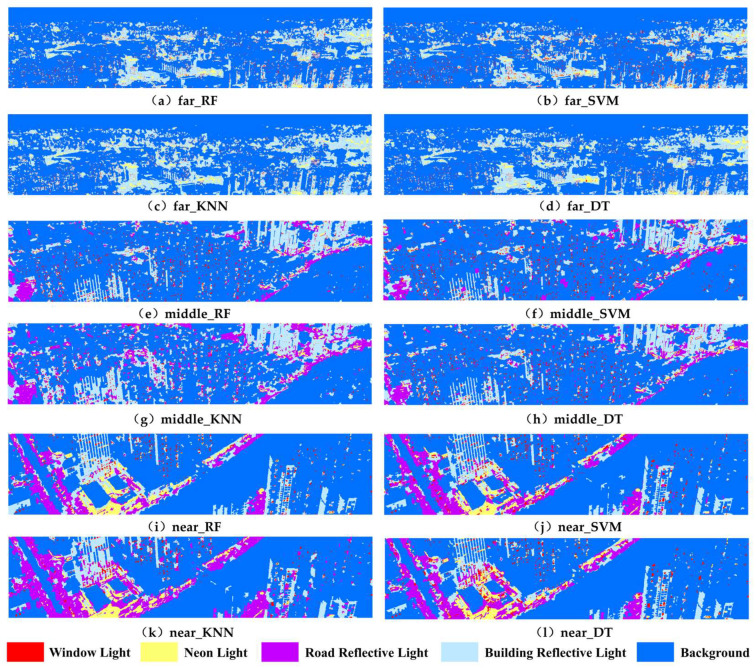
Fine-grained classification results of four machine learning algorithms for urban nighttime lights in near, middle and far view regions.

Based on the segmentation, four classification algorithms, RF, SVM, KNN and DT, were used to classify the lighting objects, and the classification results are shown in Figure 6. In general, RF had the best classification results, and its classification results reflected the distribution of various types of urban lights more accurately; in particular, the window lights and backgrounds in the near, middle and far view regions are perfectly identified. The classification results of SVM and DT were the second best, and the two types of algorithms classified window light and building reflective light better; but, compared with RF, the SVM algorithm had more sticky recognition phenomena of identifying neon light periphery as window light in the mid- and far-view regions, there was the phenomenon of identifying multiple window lights as one window light, and more misclassification of road reflective light in the near- and mid-view regions. DT had more misdistribution of window light and road reflective light than RF. The KNN had the worst classification results, with a particularly high number of misclassifications and sticky recognition phenomena, especially for road and building reflective lights.

#### 3.2.2. Accuracy Assessment of Fine Classification of Urban Nighttime Lighting

In this paper, the confusion matrices for different regions and algorithms were calculated separately based on the validation samples, and this was used to obtain the classification accuracy for each type of light, as well as the OA and kappa (Table 5, Table 6 and Table 7). Collectively, the OA of all three regions was above 80% and the kappa was above 0.75, among which the OA and kappa of the far view region were the largest, both above 90%, with the best classification effect.

The classification accuracy shows that the OA and kappa of RF were higher than those of SVM, KNN and DT in all three regions (Table 5, Table 6 and Table 7), among which the OA and kappa were the highest in the vision region with 95.36% and 0.9381, respectively. Therefore, the RF algorithm performed better than the other three algorithms in the fine classification of urban nighttime lights.

In the classification accuracy of each light type in the far view region (Table 5), the types of light with the best classification accuracy were window light and background, with neon light and building reflective light being relatively poor. The UA of all light types was higher than 87%, and the PA of all except KNN for road reflective light was lower than 85%, indicating that these four algorithms have lower misclassification rates and omission rates for all types of urban lights in the vision area.

In the classification accuracy of each light type in the middle view region (Table 6), the classification accuracy of the background was the highest, followed by the window light, and the lowest accuracy was the road reflective light. The PA and UA of each light type in the middle view region were higher than 72%, and the lowest UA was the classification accuracy of DT for neon light, which was only 72.50%, with a high misclassification rate; the lowest PA was the classification accuracy of SVM for building reflective light, which was 73.17%, with a high omission rate.

In the classification accuracy of each light type in the near view region (Table 7), the classification accuracy of background was the best, and the classification accuracy of road reflective light was the worst. The lowest result in UA was the classification result of KNN for building reflective light, only 60.32%, with a very high misclassification rate, and the highest was the classification of background by SVM, with 100% accuracy and all correct classification; the lowest in PA was the classification accuracy of KNN for road reflective light, which was only 73.85% with a high omission rate, and the highest was the classification of RF for background with 100% accuracy and no omission.

In summary, the classification accuracy of each light type is ranked as follows: background > window light > neon light > building reflective light > road reflective light. The classification accuracy of background and window light was the highest, and the classification accuracy of road reflective light was the lowest.

### 3.3. Feature Contribution Analysis

**Figure 7:**
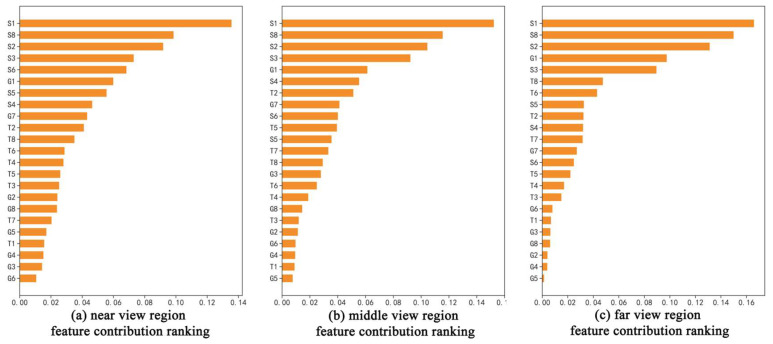
Feature contribution ranking.

To further explore which specific features had the greatest degree of influence on the fine classification of nighttime urban lighting, the RF algorithm was used to obtain the ranking of feature contributions in different regions (Figure 7). Among the three regions, the feature with the highest contribution was the spectral feature, and the total contribution of the spectral feature was 59.63% in the near view region, 63.49% in the middle view region and 66.09% in the far view region; the proportion of the region increased gradually from near to far, indicating that the smaller the classification object was, the higher the contribution of the spectral feature was. Among them, Mean_Red (S1), Mean_Green (S2) and Brightness (S8) account for the top three of the three regional features, accounting for 32.19%, 36.92% and 44.34% from near to far, respectively, indicating that the cities are more sensitive to the red and green bands at night compared with the blue band. The contribution of geometric features was the smallest, 17.43% in the near view region, 14.77% in the middle view region and 14.67% in the far view region, and the contribution decreased gradually from near to far, indicating that the smaller the classification object was, the smaller the proportion of geometric features was, and the same went for textural features. It can be seen that the contribution of spectral features was the largest and the contribution of geometric features was the smallest in the nighttime urban lighting images captured by UAVs, and the smaller the classification object was, the larger the contribution of spectral features was, while the contribution of geometric and textural features was smaller.

### 3.4. Offsite Application Comparison

**Figure 8:**
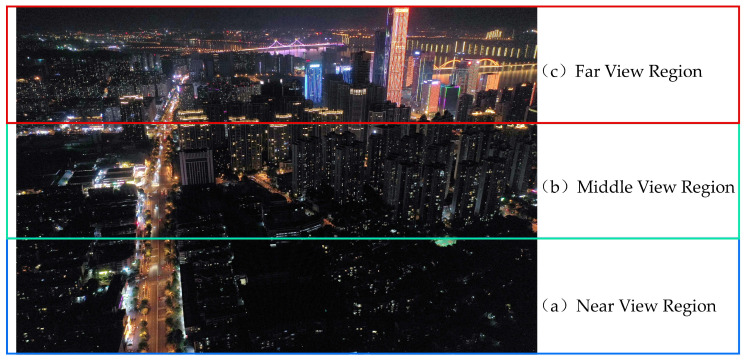
Guanshaling night lighting image and region division.

The research area selected in this paper is a typical mature community in urban construction in Changsha. Community buildings are mainly high-rise buildings, dense buildings, and rich in nighttime lighting types, providing a very good research area for fine classification of urban lighting at night. However, there are differences in the characterization of nighttime urban lights on UAV images in different regions. In order to explore the performance of this paper’s method in other areas of Changsha City and further verify the feasibility and accuracy of the object-oriented method in the fine classification of static monocular tilted urban nighttime light images captured by UAV, the old urban area of Changsha City, represented by Guanshaling (Figure 8), was selected as the research area for the comparison of different applications in this paper. The RF with the highest classification accuracy in the Meixi Lake area was used as the classification algorithm, and the method of this paper was used for classification.

As can be seen from the classification accuracy (Table 8), the classification accuracy in Guanshaling was similar to that of Meixi Lake. The overall classification accuracies of the three regions of near, middle and far view of Guanshaling were all above 85%, and the kappa were above 0.8. The feasibility and accuracy of the classification method of this paper in the fine classification of static monocular tilted urban nighttime light images captured by UAV were verified in different study areas.

## 4. Discussion

The classification accuracy shows the highest classification accuracy in the far view region, because the various light types in the far view region are smaller, the internal features of the object are more homogeneous and fluctuate less, and it is easier to distinguish between different types of lights. At the same time, in the tilt image, the more distant the region from the shooting point, the higher the probability that the road reflective light is blocked. The selected far view region in this paper directly without the road reflective light as a light type, correspondingly reducing part of the misclassification phenomenon. The classification accuracy of the middle view region was the lowest, the OA of the RF algorithm was 89.9%, and the kappa was 0.8732. Compared with the near view region, the object resolution of the middle view region was lower, and in the upper-right position of the middle view region (Figure 6), various lights were interspersed together, which increased the difficulty of classification. Additionally, the results from the offsite application comparison show that the accuracy of the middle view region was higher than that of the near view region, because the light types in the middle view region in the offsite application were not as complex as in Figure 6, so the classification accuracy of urban lights in different regions was ultimately affected by both the image resolution and the complexity of lights.

Our study indicated that RF had the highest classification accuracy in the three regions (Table 5, Table 6 and Table 7), which is in line with the findings of many classification studies of daytime UAV images [12,35,36,37]. The RF algorithm integrates multiple decision trees with good high-dimensional data processing capability and can effectively avoid noise interference. In this paper, more types of lighting features were selected, so the RF classification algorithm with better high-dimensional data processing capability had better classification results, especially in the near-field region where the accuracy difference with other classifiers was the largest, and OA was 3.92% higher than that of the second-ranked SVM. SVM had the second highest classification accuracy, and, as can be seen from Table 5, the OA difference with RF in the far-field region was the smallest at 1.79%, indicating that SVM had some advantages in dealing with small target object recognition, but was less capable of handling high-dimensional data and resisting noise than RF. KNN and DT had the lowest classification accuracy because KNN is a lazy classifier, and the nighttime light objects in this paper are more complex and more prone to misclassification and omission of lights; similarly, DT has a simple calculation process, which is difficult to cope with the task of fine classification of urban nighttime light types.

In the fine classification results of each light type (Table 5, Table 6 and Table 7), there were significant differences in the classification accuracy of each type of light. Taking the classification results of RF in the near view region as an example, the UA of road reflective lighting was only 85.71%, and the UA of other light types were above 90% (Table 7). The reasons for this phenomenon mainly include the following three points: (1) different types of street lights lead to huge differences within road reflective lighting; (2) more noise points in road reflective lighting, such as cars, pedestrians and traffic signs such as zebra lines; (3) vehicles and store lights also lead to different characteristics of road reflective lighting. Therefore, the classification accuracy and results of road reflective lighting were poor in both the near and medium view regions. The lighting types with the best classification results were window lighting and background, as both have more homogeneous internal properties and do not fluctuate much.

This study shows that spectral features occupy a greater contribution in the classification of urban nighttime low-brightness images, which is in line with the conventional perception that the brightness increases almost gradually from low to high from background, building reflected light, road reflected light, window light to neon light. The contribution of spectral features in the near, middle and far view regions was above 59%, and the proportion was gradually increasing, mainly because the smaller the classification object was, the less obvious its texture and geometric features were. For example, in the far view region, the window light directly became a small rectangle with uniform size and shape, which greatly reduced the separability of the window light in terms of texture and geometric attributes.

## 5. Conclusions

In this paper, based on static monocular tilted urban nighttime light images captured by UAVs, we qualitatively and quantitatively analyze the classification results and accuracy of different machine learning algorithms for common urban lights, and analyze the contribution of various types of features in fine classification, using an object-oriented classification method. The main conclusions are as follows:

(1) The resolution of the static monocular tilt image captured by the UAV is not a fixed value, and the variable scale problem will affect the subsequent segmentation and classification operations. In this paper, we propose the solution of dividing the image into three regions equally according to the visual direction, which can alleviate the variable scale problem, and use the segmentation method combining Canny edge detection and the multiresolution segmentation algorithm to alleviate the over-segmentation phenomenon generated when using multiresolution segmentation algorithm only.

(2) By comparing the accuracy of four classification algorithms, RF, SVM, KNN and DT, it was found that RF has the highest classification accuracy, including the highest classification accuracy in the far view region, with OA of 95.36% and kappa of 0.9381. SVM is the second, and KNN and DT have the worst classification accuracies, indicating that the RF model based on the integrated learning algorithm is more suitable for object-oriented UAV urban nighttime lights fine classification. Additionally, the OA of RF in off-site applications were above 85%, and kappa were above 0.8, which further verifies the feasibility and portability of the method in this paper.

(3) In the fine classification results of light types, window light and background have the highest classification accuracy, and their UA and PA in the RF classification model were above 93%, neon light and building reflective light were second, and road reflective light had the lowest accuracy.

(4) In the feature importance ranking, the contribution rate of spectral features was the highest, with the three regions of near, middle and far view all above 59%, among which the highest was 66.09% in the far view region; the contribution rate of geometric features was the lowest, with the three regions of near, middle and far view all below 18%, among which the lowest was 14.67% in the far view region. It indicates that the smaller the classification object is, the higher the sensitivity to spectral features and the lower the sensitivity to texture and geometric features in urban nighttime lighting images.

This paper demonstrates that the classification method combining object-oriented and traditional machine learning algorithms is applicable to the fine classification of urban nighttime light images captured by UAV static monocular tilt. This study makes up for the lack of traditional nighttime lighting at the vertical level of cities and smaller-scale urban studies, and provides a practical research idea for using finer-scale urban nighttime lighting images for related studies. Overall, the results of urban nighttime lighting classification obtained by this method can meet the needs of further research and provide methodological and data support for nighttime urban research. However, there are some parts to be improved in this paper: for example, only visible images are used, and hyperspectral and LiDAR images are not involved; for the variable scale problem, it is only solved by simply dividing it into three regions, and no finer solution is proposed. Therefore, future research will further improve these two parts.

## Figures and Tables

**Table 1:** UAV parameter settings.

Sensor	Flight Altitude	Resolution	Tilt and Turn	ISO	Aperture Size	Exposure
CMOS	300 m	0.25472 × 3648	−20°	800	2.8	1/15

**Table 2:** Feature information and number.

Feature Category	Feature Name (Number)	Number of Features
Spectral features	Mean_Red (S1), Mean_Green (S2), Mean_Blue (S3), SD_Red (S4), SD_Green (S5), SD_Blue (S6), Max_diff (S7), Brightness (S8)	8
Textural features	GLCM_Correlation (T1), GLCM_Homogeneity (T2), GLCM_Contrast (T3), GLCM_StdDev (T4), GLCM_Ang_2nd (T5), GLCM_Dissimilarity (T6), GLCM_Entropy (T7), GLCM_Mean (T8)	8
Geometric features	Area (G1), Length/Width (G2), Compactness (G3), Density (G4), Main_direction (G5), Roundness (G6), Shape_index (G7), Asymmetry (G8)	8

**Table 3:** Number of training and testing samples for different light types.

Light Category	Total Samples	Training Samples	Validation Samples
Window Light	550	385	165
Neon Light	530	371	159
Road Reflective Light	300	210	90
Building Reflective Light	530	371	159
Background	530	371	159

**Table 4:** Optimal segmentation parameters for near, middle and far view regions.

Region Name	Scale Parameter	Shape Factor	Compactness
Far view region	32	0.2	0.5
Middle view region	42	0.4	0.7
Near view region	52	0.3	0.8

**Table 5:** Far view region classification accuracy table (%).

Light Category	RF		SVM		KNN		DT	
	UA	PA	UA	PA	UA	PA	UA	PA
Window Light	97.30	98.63	95.95	92.21	100.0	96.10	90.54	98.53
Neon Light	93.24	97.18	90.54	100.0	89.19	100.0	93.24	93.24
Building Reflective Light	95.46	88.73	90.91	85.71	93.94	81.58	95.46	86.30
Background	95.46	96.92	96.97	96.97	87.88	95.08	95.46	96.92
OA	95.36		93.57		92.86		93.57	
Kappa	0.9381		0.9142		0.9047		0.9143	

**Table 6:** Middle view region classification accuracy table (%).

Light Category	RF		SVM		KNN		DT	
	UA	PA	UA	PA	UA	UA	PA	UA
Window Light	93.48	95.56	95.65	86.27	89.13	83.67	84.72	79.59
Neon Light	90.00	85.71	85.00	91.89	77.50	83.78	72.50	78.38
Road Reflective Light	80.85	90.48	74.47	94.59	85.11	80.00	78.72	80.44
Building Reflective Light	88.24	78.95	88.24	73.17	76.47	81.25	82.35	75.68
Background	100.0	100.0	100.0	96.88	96.77	100.0	93.55	100.0
OA	89.90		87.88		84.85		81.82	
Kappa	0.8732		0.8480		0.8090		0.7712	

**Table 7:** Near view region classification accuracy table (%).

Light Category	RF		SVM		KNN		DT	
	UA	PA	UA	PA	UA	UA	PA	UA
Window Light	93.02	93.02	95.35	75.93	95.35	82.00	83.72	75.00
Neon Light	91.67	88.00	81.25	86.67	81.25	90.70	75.00	76.60
Road Reflective Light	85.71	88.89	80.36	88.24	85.71	73.85	83.93	82.46
Building Reflective Light	90.48	89.06	84.13	91.38	60.32	84.45	80.95	87.93
Background	97.78	100.0	100.0	95.74	97.78	84.62	97.78	97.78
OA	91.37		87.45		82.35		83.92	
Kappa	0.8916		0.8428		0.7793		0.7983	

**Table 8:** Classification accuracy of Guanshaling.

Accuracy Index	Near View Region	Middle View Region	Far View Region
OA	86.67%	91.11%	93.02%
Kappa	0.8320	0.8889	0.9124

## Data Availability

Not applicable.
